# Cloud-Based Brain Magnetic Resonance Image Segmentation and Parcellation System for Individualized Prediction of Cognitive Worsening

**DOI:** 10.1155/2019/9507193

**Published:** 2019-01-29

**Authors:** Ryo Sakamoto, Christopher Marano, Michael I. Miller, Constantine G. Lyketsos, Yue Li, Susumu Mori, Kenichi Oishi, Alzheimer's Disease Neuroimaging Initiative ADNI

**Affiliations:** ^1^Department of Radiology, Johns Hopkins University School of Medicine, Baltimore, MD, USA; ^2^Department of Radiology, Kyoto University School of Medicine, Kyoto, Japan; ^3^Department of Psychiatry and Behavioral Sciences, Johns Hopkins Bayview and Johns Hopkins University, Baltimore, MD, USA; ^4^Division of Geriatric Psychiatry, University of Maryland School of Medicine, Baltimore, MD, USA; ^5^Center for Imaging Science, School or Engineering, Johns Hopkins University, Baltimore, MD, USA; ^6^AnatomyWorks, LLC, Baltimore, MD, USA; ^7^F. M. Kirby Research Center for Functional Brain Imaging, Kennedy Krieger Institute, Baltimore, MD, USA; ^8^Alzheimer's Disease Neuroimaging Initiative Study, USA

## Abstract

For patients with cognitive disorders and dementia, accurate prognosis of cognitive worsening is critical to their ability to prepare for the future, in collaboration with health-care providers. Despite multiple efforts to apply computational brain magnetic resonance image (MRI) analysis in predicting cognitive worsening, with several successes, brain MRI is not routinely quantified in clinical settings to guide prognosis and clinical decision-making. To encourage the clinical use of a cutting-edge image segmentation method, we developed a prediction model as part of an established web-based cloud platform, MRICloud. The model was built in a *training dataset* from Alzheimer's Disease Neuroimaging Initiative (ADNI) where baseline MRI scans were combined with clinical data over time. Each MRI was parcellated into 265 anatomical units based on the MRICloud fully automated image segmentation function, to measure the volume of each parcel. The Mini Mental State Examination (MMSE) was used as a measure of cognitive function. The normalized volume of 265 parcels, combined with baseline MMSE score, age, and sex were input variables for a Least Absolute Shrinkage and Selection Operator (LASSO) regression analysis, with MMSE change in the subsequent two years as the target for prediction. A leave-one-out analysis performed on the training dataset estimated a correlation coefficient of 0.64 between true and predicted MMSE change. A receiver operating characteristic (ROC) analysis estimated a sensitivity of 0.88 and a specificity of 0.76 in predicting substantial cognitive worsening after two years, defined as MMSE decline of ≥4 points. This MRICloud prediction model was then applied to a *test dataset* of clinically acquired MRIs from the Johns Hopkins Memory and Alzheimer's Treatment Center (MATC), a clinical care setting. In the latter setting, the model had both sensitivity and specificity of 1.0 in predicting substantial cognitive worsening. While the MRICloud prediction model demonstrated promise as a platform on which computational MRI findings can easily be extended to clinical use, further study with a larger number of patients is needed for validation.

## 1. Introduction

Cognitive disorders and dementia, heterogeneous conditions that include various brain diseases, are common in old age. Regardless of the diagnosis, one of the greatest stressors for dementia patients and caregivers is future uncertainty surrounding change progression of their condition. In clinical care settings, including memory clinics, medical providers make the best possible clinical diagnosis to inform the patient and caregivers about future progression, the type of care needed, problems that might occur in the future, and how to prevent or ameliorate these problems [1]. Accurate, individualized ways to predict cognitive progression, ones that could easily be applied in typical clinical settings, would be a great advance with huge clinical benefit for patients and caregivers.

Many neuroanatomical predictors of cognitive decline have been identified in previous studies of cognitively normal or cognitively impaired elders. These best imaging predictors involve atrophy in selected brain structures, such as areas of the mesial and lateral temporal lobes, the posterior cingulate, the orbitofrontal gyri, and white matter hyperintensity [2–4]. Non-neuro-anatomical predictors [5], such as age—with younger having worse prognosis [6], baseline cognitive function [7–9], and vascular risk factors [10], are well established. However, individualized prediction (*precision medicine*) of future decline based on individual variables is difficult because each factor only weakly correlates with the outcome, and often overlaps and interacts with other factors. Integration of these variables is, thus, essential for accurate prediction [11–14]. For such integration, important features that relate to disease progression must be extracted and recorded in a standardized quantitative manner. However, a widely available platform that could enable such quantification and integration has not been established.

In an initial attempt to develop clinically useful individualized prediction, we used a web-accessible, cloud-based platform MRICloud (https://mricloud.org/) [15] to achieve image standardization, quantification, and cross-variable integration. An integrative cloud platform is the critical enabling technology for the proposed prediction as it requires a large amount of atlas resources and intensive computation. While previous research has succeeded in predicting cognitive outcomes for research populations [3, 13, 16, 17], one of the greatest barriers to clinical application is fragmentation of analysis pipelines, which require aggregation of various tools with different capabilities on different platforms to extract a prediction value from a single image. MRICloud provides for seamless integration of whole-brain segmentation and subsequent prediction. Moreover, it enables users to develop their own applications using its programming interfaces. For example, users can implement their own data processing and analysis pipeline in MRICloud. This flexibility is particularly important in the era of machine-learning and artificial intelligence where rapid improvement, advancement, and innovation are expected.

Heterogeneity in symptoms and comorbidities encountered in clinical practice, compared to research populations with strict inclusion and exclusion criteria, is one of the major causes for which research discovery has not made its way to clinical application, as pointed out by [14]. In the era of “big data” science, the application of a trained algorithm to *real-world data* is necessary to validate its usefulness in day to day clinical practice. The MRICloud platform is promising for such a validation study since it enables the collection of raw MRIs from all over the world through its web interface. To test this concept, we applied a prediction model, developed on a research cohort, to a real clinical population of cognitively impaired patients to investigate the applicability of the prediction model to a heterogeneous clinical setting.

## 2. Methods

### 2.1. Training Dataset

Data used in the preparation of this article were obtained from Alzheimer's Disease Neuroimaging Initiative (ADNI) database (https://adni.loni.usc.edu). ADNI was launched in 2003 as a public-private partnership, led by Principal Investigator Michael W. Weiner, MD. The primary goal of ADNI has been to test whether serial magnetic resonance imaging (MRI), positron emission tomography (PET), other biological markers, and clinical and neuropsychological assessment can be combined to measure the progression of mild cognitive impairment (MCI) and early Alzheimer's disease (AD). The data were analyzed anonymously, using publicly available secondary data. Therefore, no specific ethics approval was required for this work.

A total of 402 individuals available from the ADNI-1 database, with corresponding baseline MRIs and two years of follow-up data, were analyzed (AD = 75, MCI = 176, and cognitively normal individuals = 151). Structural MRIs were acquired from 1.5T scanners with a protocol individualized for each scanner, as defined in http://adni.loni.usc.edu/. The MRIs were downloaded from https://ida.loni.usc.edu/ in NiFTI formats, with geometry distortion correction and B1 correction. The demographics and characteristics of the study population are in Table 1.

### 2.2. Test Dataset

Imaging and clinical data acquired as part of clinical care in the Johns Hopkins Memory and Alzheimer's Treatment Center (MATC) located at the Bayview Medical Center were used as the test dataset. The MATC dataset consists of patients with memory problems, self-referred or referred by other physicians, evaluated in an outpatient memory disorders clinic. The creation and use of the database occurred under oversight by the Johns Hopkins Institutional Review Board, which provided waiver of consent, as the data were all collected in clinical care [18]. This patient cohort was heterogeneous, with various etiologies and levels of severity, and represented diverse people with memory problems. A total of 17 patients with baseline MRIs and two years of clinical follow-up data were analyzed. MRI scans, acquired for clinical care only, followed the ADNI protocol: a three-dimension (3D), magnetization-prepared, rapid gradient-echo sequence, with a repetition time of 2300 ms, an echo time of 2.98 ms, and a voxel resolution of 1 × 1 × 1 mm, scanned on a 3T scanner (SIEMENS Vario). The demographics and characteristics of the selected population are given in Table 2.

### 2.3. Image Processing

A multiatlas label fusion method in which an entire brain is automatically parcellated into 265 anatomical units [19] was applied to the MRIs. This is a fully automated method that is open to public use through our website (https://mricloud.org/). All MRIs were bias-corrected and linearly aligned to the JHU-MNI atlas [20] space. Atlases were warped to the linearly aligned subject image using Large Deformation Diffeomorphic Metric Mapping (LDDMM) [21], followed by application of the multiatlas fusion algorithm [19]. The JHU T1 Geriatric Multi-Atlas Inventory V5 [22, 23], designed for older patient populations with potential brain atrophy, was used as a set of atlases. The volume of each parcel was measured and normalized based on the whole-brain volume.

### 2.4. Neuroimage Features

**Figure 1:**
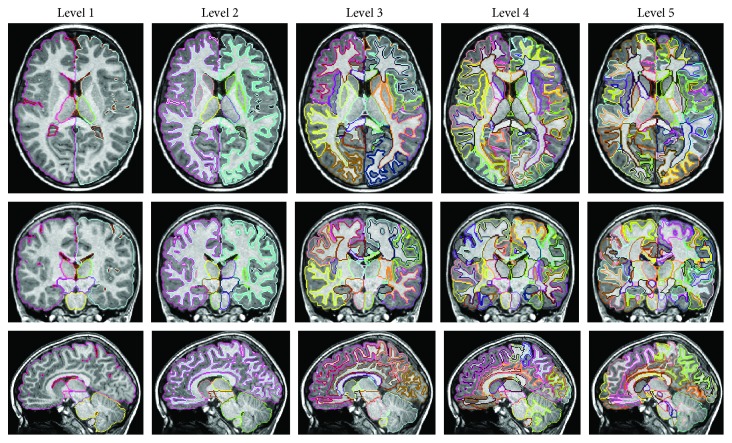
Hierarchical relationships of 254 structures defined in the MRICloud.

One of the challenges of atlas-based image analysis is the granularity of the anatomical parcellation map used to quantify brain MRI [24–26]. The statistical power to characterize anatomical features related to cognitive decline is maximized when the size and shape of the parcel exactly follow the pathological locations that determine the prognosis. If there is an *a priori* hypothesis about the distribution of the pathologic tissues, predefinition of the size and shape of parcels can follow the hypothesis. However, impaired anatomical structures and spatial distribution depend on the disease severity seen in the brain MRI, which are unknown prior to the analysis. To account for the heterogeneity of this patient population, we applied a tool that can flexibly change the granularity level based on the hierarchical relationships of 254 structures defined in our atlas [27], in which the 254 structures were assigned a hierarchical relationship based on their ontological relationship [28, 29]. This relationship consists of five hierarchical levels that were named: Level 1 (11 parcels); Level 2 (17 parcels); Level 3 (36 parcels); Level 4 (54 parcels); and Level 5 (254 parcels) (Figure 1). The volumes of all structures in each level were obtained.

### 2.5. Non‐Image Features

Many non‐image predictors of cognitive decline have been identified in previous studies of cognitively normal or cognitively impaired elders. These can be summarized into seven major categories [5]: sociodemographics; clinical characteristics; cognitive or neuropsychological features; behavioral or psychological factors; cardiovascular risk factors; genetics; and biological markers. Although inclusion of all these factors into the prediction model might maximize the prediction accuracy, obtaining and recording such information comprehensively in a structured way is not a feasible standard for the vast majority of clinical care settings. We attempted to balance this by focusing on (preselecting) predictor variables available in pretty much all clinical care setting: age, sex, and MMSE score at the time of the MRI scan.

### 2.6. Prediction Algorithm

A Least Absolute Shrinkage and Selection Operator (LASSO) regression analysis [30, 31] was estimated to predict worsening in cognition two years after the baseline. The LASSO was chosen to address the multicollinearity problem and to identify important predictors. The MMSE measured cognitive function, and change in MMSE was set as the target for prediction. Among various cognitive measures, the MMSE was chosen because it is most commonly measured in patients who visit memory clinics and can easily be also measured in primary care settings. The LASSO regularization weight parameter *λ* was selected in order to minimize a mean squared prediction error between the measured and the predicted ΔMMSE obtained by leave-one-out cross validation. The normalized volume of anatomical units, baseline MMSE, age, and sex were used as a set of variables.

### 2.7. Validation

To validate the prediction algorithm within the ADNI population, a leave-one-out analysis was performed to investigate the correlation between the measured and the predicted MMSE change. The analyses were performed based on level of granularity (Levels 1–5) and all levels combined. The predictor model that provided the best correlation with cognitive outcome in ADNI data was then applied to the clinical population.

There is considerable debate about the disease-specific speed of cognitive worsening [32, 33], although a meta-analysis suggested a similar pace of cognitive decline in two of the most common diagnoses of dementias, AD and dementia with Lewy bodies [34]. Faster cognitive decline is seen in autopsy-confirmed frontotemporal dementia (FTD) compared to AD [33]. Within the AD population, there are at least two distinctive types of disease progression, slow and rapid [7, 35–37]. Since we aimed to identify rapid progressors in the clinical cohort of mild cognitive impairment or mild dementia (MMSE ≥ 23) at baseline, and majority of the ADNI training data fall into this category (Table 1), we used MMSE decline of ≥4 points after two years to define substantial cognitive worsening, according to the reported average decline in MMSE score over two years (3.3 points decline in MMSE score) in the mild AD population with a baseline MMSE score of 23 [38]. To investigate the accuracy to predict substantial cognitive worsening, ROC analysis was performed. The cutoff of the predicted MMSE change that maximized the Youden index [39] was adopted as the threshold to predict substantial cognitive worsening (true MMSE decline of ≥4 points). The glmnet package [31] implemented in R software [http://cran.r-project.org, R Core Team, version 3.2.3] was used for the LASSO regression analysis and the ROC curve analysis.

### 2.8. Implementation of the Prediction Model into the MRICloud

The MRICloud provides a cloud-based architecture for neuroimage analysis tools through the web. It has three components: storage; computation; and applications. It also provides visualization applications and enables users to develop their own application with the application programming interfaces (API). It provides low-barrier access to the algorithms and tools and accommodates high throughput, as well as parallel computation, to render intensive computations tractable. The prediction model developed was integrated into the MRICloud using the API, which allows users to upload their own image and non‐image data to predict the MMSE change.

### 2.9. Application of the Prediction Algorithm to the Clinical Population

The prediction model implemented in MRICloud was applied to the clinical cohort for individualized prediction of the MMSE change over the two years after the initial evaluation. A correlation between true and predicted MMSE change, as well as sensitivity, specificity, positive predictive value (PPV), and negative predictive value (NPV) to predict substantial cognitive worsening were calculated.

## 3. Results

### 3.1. Generation of Prediction Model

**Figure 2:**
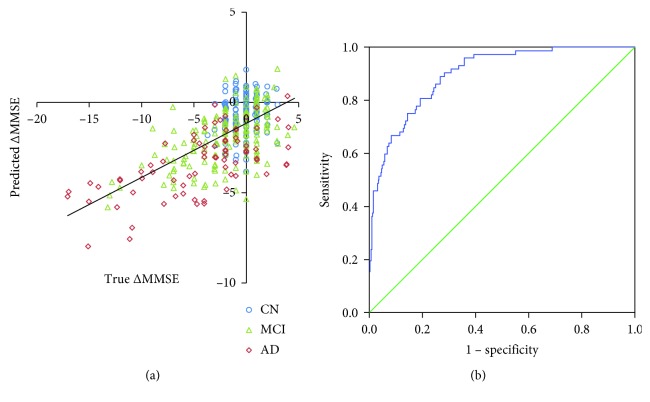
(a) Scatter plot showing the relationship between true and predicted MMSE change based on the training dataset. (b) ROC analysis showing the relationship between sensitivity and specificity to predict substantial cognitive worsening. CN: cognitively normal, MCI: mild cognitive impairment, and AD: Alzheimer's disease.

A leave-one-out analysis of the training dataset estimated a larger correlation coefficient between true and predicted MMSE change at the highest granularity level (level 5, *R* = 0.635) and at all levels combined (*R* = 0.636) (Figure 2(a)) compared to the lower granularity levels (Level 1, *R* = 464, Level 2 = 0.489, Level 3 = 0.527, and Level 4 = 0.599). The ROC analysis (Figure 2(b)) using an applied regression model estimated areas under the curve (AUC) of 0.898 (Level 5, 95% CI: 0.862–0.935) or 0.899 (all levels combined, 95% CI: 0.862–0.936) for the prediction of substantial cognitive worsening. Therefore, the prediction model using local brain volumes of all granularity levels combined was used in later analyses. The optimal cut of value for the prediction of substantial cognitive decline, calculated from the ROC curve, was a predicted MMSE decline of −1.9, which demonstrated sensitivity of 0.847, specificity of 0.779, PPV of 0.455, and NPV of 0.959 for substantial cognitive worsening. The mean absolute difference between the true and predicted MMSE change was 2.0 (SD = 2.0).

### 3.2. Contribution of Each Factor to the Prediction

**Figure 3:**
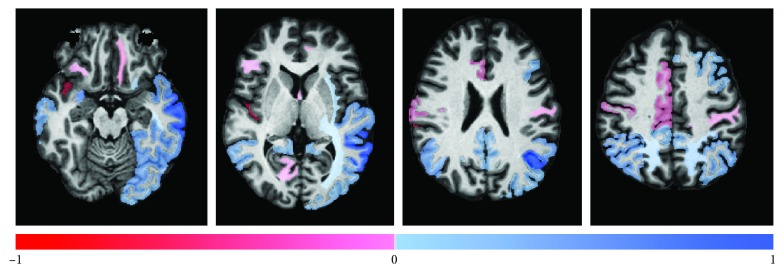
Regression coefficients of the selected anatomical structures are color-coded (blue: positive regression coefficient and red: negative regression coefficient) and overlaid on the JHU-MNI atlas.

Among 402 ADNI participants, 83 (21%) were rapid progressors (Table 1). Thirty-one factors (volumes of 29 anatomical areas, baseline MMSE score, and age, but no sex) were selected through the LASSO regression analysis using all granularity levels combined and built into the final prediction model. The standardized regression coefficients for each of the image and non‐image factors are in Table 3. Regression coefficients for selected anatomical structures are color-coded and overlaid on the JHU-MNI atlas (Figure 3). Atrophy in the bilateral middle temporal gyri, the claustrum complex, the superior parietal white matter, expansion of the Sylvian fissure, and the lower baseline MMSE score and younger age predicted greater likelihood being a rapid progressor by MMSE change score.

### 3.3. Implementation of the Model into the MRICloud

**Figure 4:**
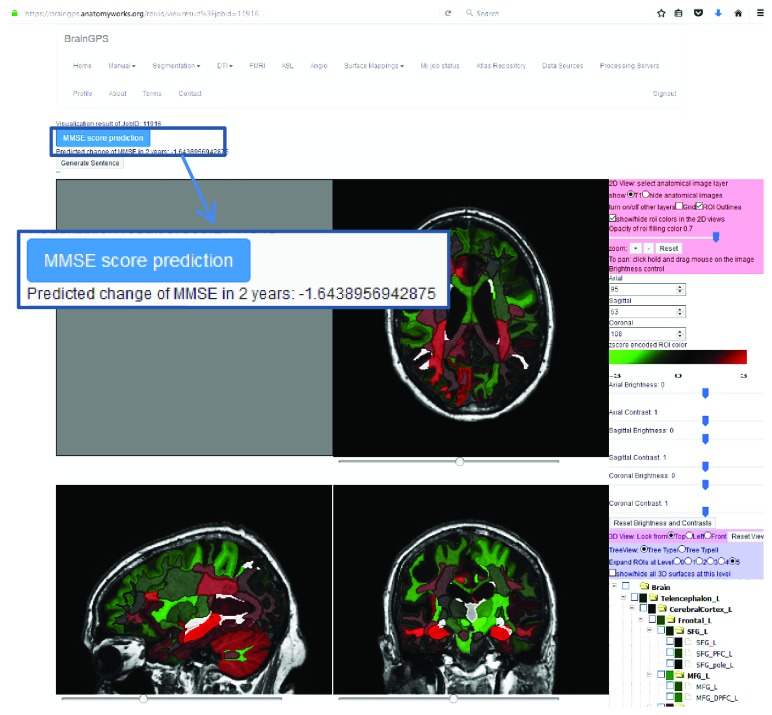
Screenshot of the MMSE score prediction function implemented in the BrainGPS module of the MRICloud. The module allows users to submit their own high-resolution, 3D, T1-weighted images with the age and MMSE score at scan. The module provides a color-coded z-score map of the local volume (lower left) as well as the predicted ΔMMSE (magnified view in the blue rectangle).

The model was then implemented into the MRICloud. This enables external users to obtain access to the prediction model for additional validation. Users can visit the website (https://www.mricloud.org) to log in to the “BrainGPS” module and then select the “T1 segmentation” tool listed in the upper row. This allows users to upload their own ADNI-compatible, high-resolution, 3D-MPRAGE images, MMSE score, and age at scan to obtain the predicted ΔMMSE in two years (Figure 4).

### 3.4. Application to the Clinical Population

**Figure 5:**
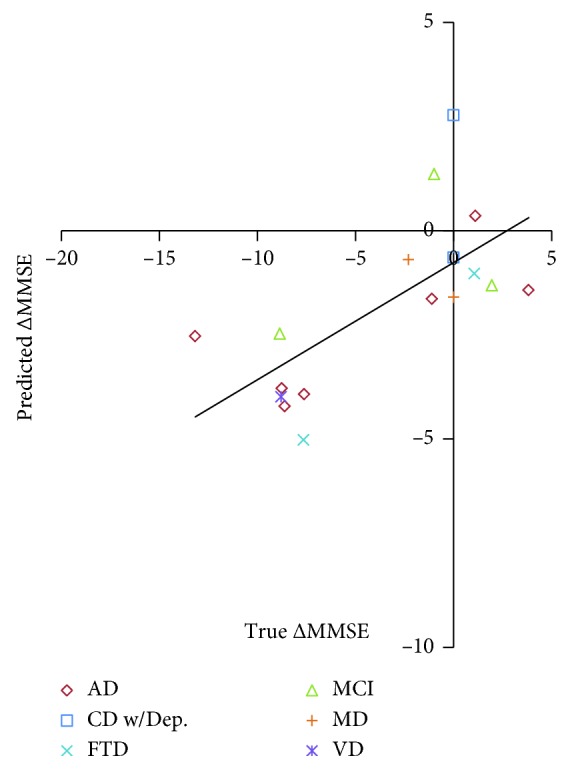
Results of the fully automated MMSE change prediction using the BrainGPS module of the MRICloud, compared to the true MMSE change. AD: Alzheimer's disease, MCI: mild cognitive impairment, CD w/Dep: nonspecific cognitive disorder with depression, FTD: frontotemporal dementia, MD: mixed dementia, and VD: vascular dementia.

Among 17 MATC patients, 7 (41%) were rapid progressors (Table 2). A graph showing the correlation (*R* = 0.69) between the actual and predicted MMSE change is shown in Figure 5. All patients with substantial cognitive worsening were accurately predicted when a predicted MMSE decline of −1.9 was applied. The mean absolute difference between the true and predicted MMSE change was 3.4 (standard deviation = 2.5).

## 4. Discussion

### 4.1. Generation of the Prediction Model

In current clinical practice for patients with cognitive disorders, the main role of structural MRI is to exclude causes such as neoplasms, hydrocephalus, trauma, or ischemic disease that are clearly visible on a scan. In clinical research, MRI is used as one of the measures of neurodegeneration caused by AD pathology [40]. Our results suggest a substantial contribution of local neuroanatomical changes to predict cognitive worsening in addition to non‐image features. The prediction model trained by LASSO indicated younger age and lower baseline cognitive function were related to faster deterioration in cognitive function, consistent with prior studies [7, 41]. The final model did not include sex as a predictor, suggesting that sex has little effect on rate of cognitive decline, as expected [42]. Since our goal was to investigate a robust index for individualized prediction from information available through clinical practice not necessarily obtained by specialists, the inclusion of only two simple non‐image factors—age and MMSE—has practical significance.

### 4.2. Brain Areas Important for the Prediction

Three anatomical areas—middle temporal gyrus, peri-Sylvian, and parietal areas—were the areas that contributed to prediction of cognitive worsening. These areas did not necessarily include anatomical structures that are known to be most affected in early AD, such as medial temporal area, but rather, overlapped with local brain areas whose volumes correlated with MMSE score, such as the temporal, the middle frontal, and the left angular and supramarginal gyri [43–45]. Previously reported correlations between enlargement of the Sylvian fissure and MMSE score in AD and MCI [46] support our findings. These results suggest that the mesial temporal atrophy, while correlated with the MMSE score at the baseline [43, 47–49] is not necessarily the best predictor of cognitive worsening in coming years.

### 4.3. Clinical Validation Study Performed on the MRICloud Platform

Although the MATC population consisted of patients with various clinical diagnoses or with mixed causes of cognitive decline, the sensitivity and specificity to predict rapid cognitive decline was comparable to that obtained from an ADNI cohort with strict inclusion and exclusion criteria. This was unexpected because we initially assumed that the prediction model trained from the ADNI dataset would be applicable only to patients with a clinical diagnosis of probable AD. One possible explanation is the existence of a spatial pattern in local brain atrophy related to an increased risk of cognitive worsening, which is less specific to AD, but rather common to various types of dementias. The applicability of the model to nonAD dementias, such as frontotemporal dementia, dementia with Lewy bodies, or vascular dementia, needs to be further investigated.

We also noted overestimated prediction of MMSE decline for patients with a low baseline MMSE score (MMSE < 23). This was probably due to the relative imprecision of MMSE in measuring cognitive decline in demented patients [50]. This explains the results of the ROC curve analysis, in which the cutoff predicted MMSE change of −1.9 was higher than the true actual change in MMSE of −4. Therefore, care should be taken when the predicted change is less than −1.9; in this case, the practical interpretation should be a “MMSE change less than −4 can be predicted.”

Other limitations include the small clinical sample size, which may have involved selection bias or confounding factors, such as age. Indeed, this limitation was one of our motivations to develop an open-access platform for future validation study, in which MRIs from other institutes can be easily submitted to increase the number of test MRIs, as detailed in the next section. The method used to select the LASSO regularization parameter could also be an issue, since inappropriate selection leads to overfitting of training data; therefore, the performance measures for the training data (ADNI data) were possibly upward-biased. Application of the nested cross validation is one possibility to remain unbiased in the cross validation-based evaluation, where the parameters are selected by cross validation [17].

### 4.4. MRICloud as a Platform for Algorithm Development and Clinical Validation Studies

External and/or clinical validation of prediction models developed in research is not easy. Such models are typically published in print with only the theoretical aspects, such as functions and variables, elaborated. However, practical implementation of the models depends on the computational environment, the operating systems used, software versions, methods to extract variables, and coding of the functions, all of which have an impact on the reproducibility of the research findings. There have been efforts to distribute the open-source scripts or software packages as the solutions for effective deployment of new models. While successful, there are several limitations in this approach. First, it places a considerable amount of the burden on developers, which includes program development for cross platforms and reengineering after a version change of the operating system, efforts to redistribute after a version-update, management of users with different versions, and inquiries from other developers about the content of the source codes. The cloud-based software-as-a-service (SaaS) model has emerged as a solution to these barriers for cross-program communications, platform independence, and efficient computation strategies. The field of machine-learning and artificial intelligence is developing rapidly, and we expect algorithms that will replace LASSO, in the near future. Currently available algorithms, such as elastic net, are already demonstrating excellent performance in MRI feature selection [51]. The SaaS through API, which allows users to implement their novel image analysis algorithm to be shared and tested with users, seems to be one of the best solutions computer science can offer at this moment. The MRICloud provided a user-friendly environment to share the prediction model with external users for rigorous validation.

## 5. Conclusion

Our results indicated the potential to apply results from a study population to clinical practice, at least in a limited venue, such as the MATC, but further study with a larger number of patients is needed to characterize the features of cognitive worsening. The MRICloud provided a user-friendly environment suitable for multi-institutional clinical validation studies to predict future cognitive worsening from image and non‐image data.

## Figures and Tables

**Table 1:** Demographics and characteristics of the training dataset (ADNI1).

Diagnosis at baseline	*N*	Age	Sex (men/women)	MMSE at baseline	MMSE after 2 years	MMSE decline in 2 years	Number of patients with substantial worsening^*∗*^
CN	151	75.5 ± 5.0	80/71	29.2 ± 1.0	29.0 ± 1.2	−0.2 ± 1.3	1
MCI	176	73.4 ± 7.1	113/63	27.2 ± 1.7	25.4 ± 3.9	−1.8 ± 3.3	46
AD	75	73.8 ± 7.4	34/41	23.3 ± 2.0	19.0 ± 5.6	−4.3 ± 5.3	36
Total	402	74.9 ± 6.5	227/175	27.2 ± 2.6	25.6 ± 5.1	−1.6 ± 3.6	83

^*∗*^Substantial worsening: MMSE declines ≤ −4 within two years.

**Table 2:** Demographics and characteristics of the test dataset (MATC database).

Suspected diagnosis at baseline	*N*	Age	Sex (men/women)	MMSE at baseline	MMSE after 2 years	MMSE decline in 2 years	Number of patients with substantial worsening^*∗*^
AD	7	73.7 ± 11.0	4/1	21.9 ± 4.5	16.9 ± 8.0	−5.0 ± 5.8	4
MCI	3	82.5 ± 2.9	1/2	29.0 ± 0.8	26.3 ± 3.9	−2.7 ± 4.6	1
Others	7	69.8 ± 10.1	3/4	23.1 ± 7.0	20.6 ± 10.1	−2.6 ± 3.8	2
Total	17	73.7 ± 10.7	8/9	23.6 ± 6.0	20.0 ± 9.1	−3.6 ± 5.0	7

Others: mixed dementia, 2; vascular dementia, 1; frontotemporal dementia, 2; nonspecific cognitive disorder with depression, 2. ^∗^Substantial worsening: MMSE declines ≤ −4 within two years.

**Table 3:** List of the standardized regression coefficients for each of the image and non‐image factors, obtained from the LASSO regression analysis.

Factors	Standardized regression coefficients
Age	0.606014
Middle temporal gyrus, left (Level 5)	0.555413
Claustrum, right (Level 5)	0.33835
Baseline MMSE score	0.221676
External capsule, right (Level 5)	0.203446
Temporal lobe, left (Level 4)	0.184202
Angular gyrus, right (Level 4)	0.179487
Superior parietal white matter, left (Level 5)	0.147153
Angular gyrus, left (Level 4)	0.146566
Fimbria, left (Level 5)	0.131843
Middle temporal gyrus, right (Level 5)	0.119239
Inferior occipital gyrus, left (Level 4)	0.09018
Middle frontal gyrus, left (Level 5)	0.083102
Posterior cingulate cortex, right (Level 5)	0.080805
Posterior cingulate cortex, left (Level 5)	0.074983
Superior parietal white matter, right (Level 5)	0.068838
Middle occipital gyrus, left (Level 4)	0.060132
Superior frontal gyrus, left (Level 5)	0.052995
Fimbria, right (Level 5)	0.045444
Inferior deep parietal white matter, left (Level 4)	0.014723
Peripheral frontal white matter, right (Level 4)	−3.00*E* − 05
Inferior frontal white matter, right (Level 5)	−0.01381
Lateral frontoorbital gyrus white matter (Level 5)	−0.03149
Lingual gyrus white matter, right (Level 5)	−0.04243
Postcentral gyrus white matter, left (Level 5)	−0.07299
Postcentral gyrus, right (Level 4)	−0.08223
Fornix, right (Level 4)	−0.08814
Dorsal anterior cingulate cortex, right (Level 5)	−0.10213
Gyrus rectus white matter, left (Level 5)	−0.16791
Mammillary body, right (Level 5)	−0.25573
Sylvian fissure and temporal sulcus, right (Level 4)	−0.29769

## Data Availability

The ADNI dataset is downloadable through the website http://adni.loni.usc.edu/. MRICloud is available for registered users (https://mricloud.org/). Data used in preparation of this article were obtained from the Alzheimer's Disease Neuroimaging Initiative (ADNI) database (adni.loni.usc.edu). As such, the investigators within the ADNI contributed to the design and implementation of the ADNI and/or provided data but did not participate in the data analysis or in the writing of this report. A complete listing of ADNI investigators can be found at http://adni.loni.usc.edu/wp-content/uploads/how_to_apply/ADNI_Acknowledgement_List.pdf.
